# Oncological outcomes of extended versus standard pelvic lymph node dissection in radical cystectomy: An updated systematic review and meta‐analysis

**DOI:** 10.1002/bco2.70257

**Published:** 2026-08-02

**Authors:** Rafael Matos Vieira Gordilho, Nilo Jorge Carvalho Leão Barretto, Rodrigo Barbosa Freire Silvão, Felipe Pereira Garrido Pazos, Pedro Rodrigues Queiroz, Matheus Oliveira Figueiredo, Ricardo Andrade Freitas Souza, João Pedro Gomes da Conceição Oliva, Luma Gabriella Firme Sampaio Góes, Rafael Cintra Rosa Corrêa Oliveira, Lorena Barboza de Sousa, Felipe Pinho, Albuquerque Silva, Leonardo Marques Calazans, Wendel Souza Kruschewsky, Alexandre Azevedo Ziomkowski, Eduardo Deda Mendonça Filho, Gabriel Silva Rocha, Marcos Tobias‐Machado, Raphael Brandão Moreira

**Affiliations:** ^1^ Brazilian Institute of Robotic Surgery Salvador Brazil; ^2^ Mater Dei Hospital Salvador Brazil; ^3^ Bahiana School of Medicine and Public Health Salvador Brazil; ^4^ Section of Urologic Oncology Arnaldo Vieira de Carvalho Cancer Institute São Paulo Brazil; ^5^ São Camilo Hospital Network São Paulo Brazil

**Keywords:** bladder cancer, extended lymph node dissection, meta‐analysis, oncological outcomes, pelvic lymph node dissection, radical cystectomy

## Abstract

**Objective:**

This study aims to evaluate whether extended lymph node dissection (ePLND) improves overall survival (OS) and oncological outcomes compared with standard dissection (sPLND) in patients undergoing radical cystectomy for urothelial bladder cancer.

**Methods:**

A systematic review and meta‐analysis were conducted according to PRISMA 2020 (PROSPERO CRD420261309465). PubMed/MEDLINE, EMBASE, Cochrane Library and Web of Science were searched. RCTs and observational studies comparing ePLND and sPLND were included. The primary outcome was OS; secondary outcomes included recurrence‐free survival (RFS), cancer‐specific survival (CSS), 5‐year OS, 5‐year RFS, major complications and lymphocele. A random‐effects model was used, with sensitivity analyses and meta‐regression. Risk of bias was assessed using RoB 2 and ROBINS‐I.

**Results:**

Sixteen studies (*n* = 5960) were included. ePLND did not significantly improve OS compared with sPLND (HR = 0.86; 95% CI 0.72–1.03; *p* = 0.09; *I* ^2^ = 50%). The overall RFS analysis suggested a benefit favouring ePLND (HR = 0.74; 95% CI 0.63–0.81; *p* < 0.00001); however, this effect did not persist in analyses restricted to RCTs. No consistent benefit was observed for 5‐year OS, although recurrence was reduced. ePLND did not significantly affect major complications. Sensitivity analyses confirmed result stability.

**Conclusion:**

Higher quality evidence does not demonstrate a consistent benefit of ePLND for OS, although overall analyses show reduced recurrence. Current data do not support the routine adoption of ePLND with the sole objective of improving survival.

## 1 INTRODUCTION

Bladder cancer is one of the most common urological malignancies worldwide. In patients with muscle‐invasive disease and in selected high‐risk non–muscle‐invasive cases refractory to intravesical therapy, radical cystectomy with pelvic lymph node dissection (PLND) remains the standard treatment.^1^, ^2^, ^3^ In addition to its potential therapeutic role, PLND provides the most accurate pathological staging and plays a central role in prognostic stratification and in guiding adjuvant therapy decisions.^4^

Despite consensus regarding the need for PLND, the optimal extent of lymph node dissection—standard (sPLND) or extended (ePLND)—remains controversial.^5^, ^6^ Extended PLND includes additional nodal basins beyond standard templates and may theoretically improve oncological control through removal of occult micrometastases and more accurate staging, a phenomenon known as stage migration.^7^, ^8^, ^9^ However, it remains uncertain whether these theoretical advantages translate into a true survival benefit.^4^, ^10^, ^11^

Much of the early evidence supporting ePLND derives from observational studies suggesting improved oncological outcomes, including overall survival (OS) and recurrence‐free survival (RFS).^12^, ^13^ However, these findings are susceptible to important biases, including patient selection, surgical expertise and stage migration. Moreover, ePLND is a more complex procedure associated with longer operative time and potentially increased perioperative morbidity.^5^, ^10^

More recently, large randomized controlled trials (RCTs) have been conducted to address this question, with results that challenge the previously suggested benefit of ePLND.^6^, ^14^ In addition, several prior meta‐analyses do not fully incorporate these contemporary data, limiting their ability to provide an up‐to‐date synthesis of the evidence.^10^, ^11^

Given these uncertainties, an updated and critical evaluation of the available literature is warranted. Therefore, we conducted a systematic review and meta‐analysis to assess whether ePLND, compared with sPLND, improves survival and oncological outcomes in patients undergoing radical cystectomy for bladder cancer.

## 2 MATERIALS AND METHODS

### 2.1 Study design

We conducted a systematic review and meta‐analysis in accordance with the PRISMA 2020 guidelines.^15^ The protocol was registered in PROSPERO (CRD420261309465).

### 2.2 Search strategy and eligibility criteria

We systematically searched PubMed/MEDLINE, EMBASE, the Cochrane Library and Web of Science using a combination of MeSH and EMTREE terms related to urothelial bladder cancer and pelvic lymph node dissection. The complete search strategy is detailed in the Supporting Information.

We included studies enrolling adult patients (≥18 years) undergoing radical cystectomy for predominantly urothelial bladder cancer that directly compared extended (ePLND) and standard pelvic lymph node dissection (sPLND). Eligible designs included randomized controlled trials (RCTs) and observational studies.

We excluded preclinical studies, animal studies, duplicate publications and studies with overlapping cohorts. sPLND was defined as dissection of obturator and internal/external iliac nodes, whereas ePLND included additional templates such as common iliac and presacral nodes.

### 2.3 Study selection and data extraction

Two reviewers independently screened studies by title/abstract and full‐text review. Data were extracted independently and in duplicate using a standardized form. Extracted variables included study characteristics, patient demographics, tumour stage, use of perioperative therapies and number of lymph nodes removed. Discrepancies were resolved by a third reviewer.

### 2.4 Outcomes

The primary outcome was overall survival (OS). Secondary outcomes included recurrence‐free survival (RFS), cancer‐specific survival (CSS), 5‐year survival outcomes, recurrence patterns, major complications (Clavien–Dindo) and lymphocele formation. Analyses were stratified by study design (RCT vs non‐RCT).

### 2.5 Risk of bias and certainty of evidence

Risk of bias was assessed using the ROBINS‐I tool for non‐randomized studies^16^ and the Cochrane Risk of Bias Tool 2 (RoB 2) for randomized trials.^17^ Two reviewers performed the assessments independently, with disagreements resolved by a third reviewer.

The certainty of evidence for primary outcomes was evaluated using the GRADE approach^18^ with GRADEpro software.^19^

### 2.6 Statistical analysis

Pooled relative risks (RRs) with 95% confidence intervals (CIs) were calculated for dichotomous outcomes, and hazard ratios (HRs) were pooled for time‐to‐event outcomes.^20^, ^21^ Heterogeneity was assessed using Cochran's *Q* and the *I* ^2^ statistic. A random‐effects model was applied.^20^

Sensitivity analysis was performed using the leave‐one‐out method, and Baujat plots were used to assess study influence. Publication bias was evaluated by visual inspection of funnel plots.

Meta‐regression was conducted to explore sources of heterogeneity, including study design (RCTs vs. observational), year of publication, neoadjuvant therapy, tumour stage, follow‐up duration and number of lymph nodes removed.^22^

Analyses were performed using Review Manager 5.4 (RevMan; The Cochrane Collaboration) and R software (R Foundation for Statistical Computing, Vienna, Austria).

## 3 RESULTS

### 3.1 Study selection

**FIGURE 1:**
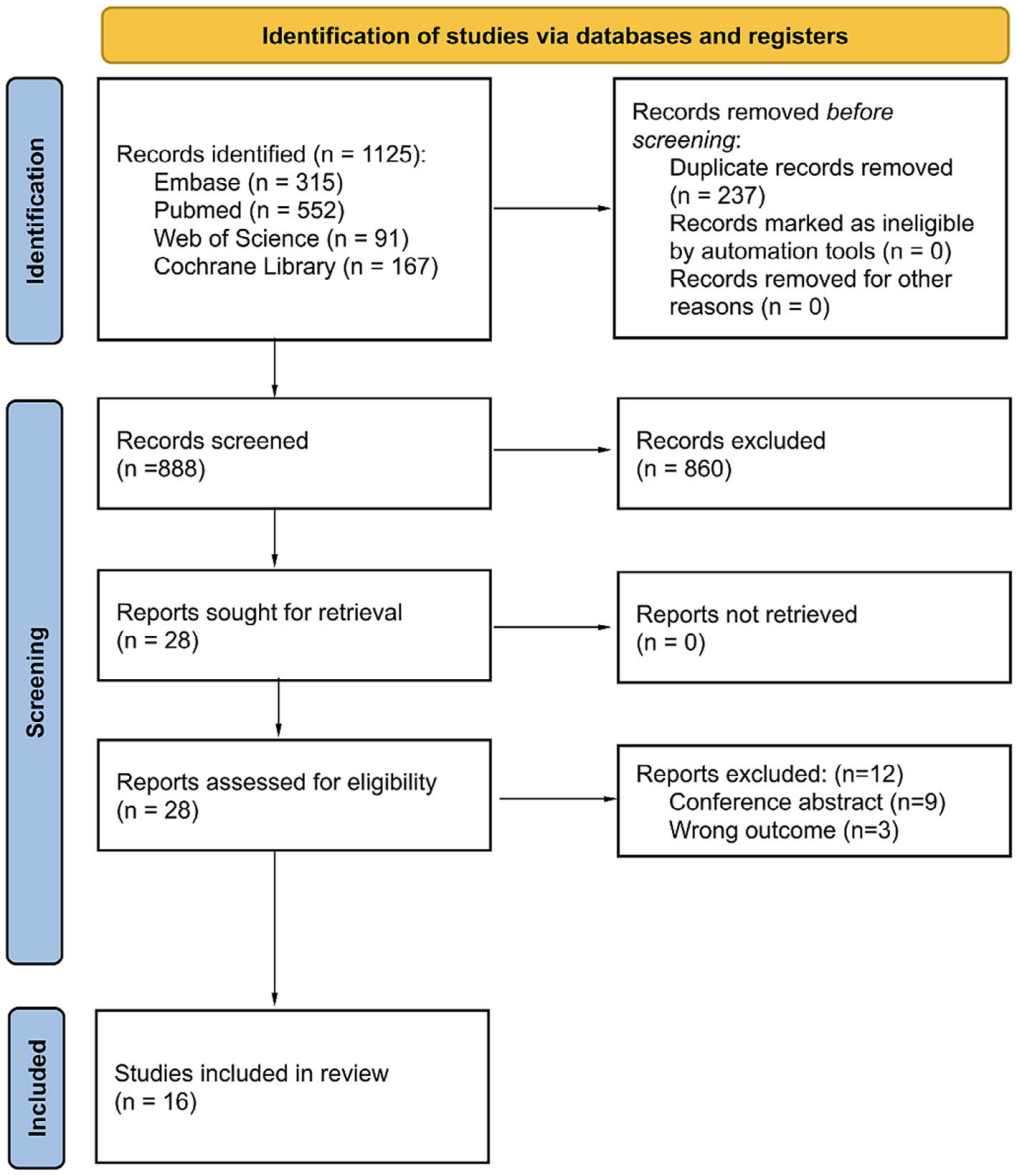
PRISMA 2020 flow diagram of study selection.

The search identified 1125 records. After duplicate removal, title/abstract screening and full‐text assessment, 16 studies were included in the quantitative synthesis (Figure 1). Detailed study characteristics are presented in Tables 1 and S1.

**TABLE 1:** Baseline characteristics of the included studies.

Author, year	Country	Study design	ePLND (*n*)	sPLND (*n*)	Age, years—ePLND	Age, years—sPLND	Male/female—ePLND (%)	Male/female—sPLND (%)	Follow‐up, months—ePLND	Follow‐up, months—sPLND	pT stage—ePLND	pT stage—sPLND	Removed LN—ePLND (*n*)	Removed LN—sPLND (*n*)
Abdi, 2015^23^	Canada	Retrospective	105	105	68.4 ± 9.0	68.7 ± 10.3	74.3/25.7	80/20	22.2 ± 21.8	20.8 ± 21.0	≤T2: 58 ≥T3: 42	≤T2: 52.4 ≥T3: 47.6	21 ± 12	9 ± 4.5
Abol‐Enein, 2011^24^	Egypt	Prospective	200	200	55 [12]	50.5 [12]	81/19	77.5/22.5	50.2 [69]	50.2 [69]	≤T2: 35.2 ≥T3: 64.8	≤T2: 30.5 ≥T3: 69.5	49 [18.7]	16 [8]
Choi, 2019^25^	South Korea	Retrospective	216	124	63.9 ± 9.5	63.4 ± 10.4	88.4/11.6	83.1/16.9	O: 40.5 ± 42.8		≤T2: 52.8 ≥T3: 47.2	≤T2: 52.4 ≥T3: 47.6	24.3 ± 11.9	14.3 ± 8.2
D'Andrea, 2019^26^	Interational	Retrospective	34	200	67 ± 9.1	67.3 ± 9.7	73.5/26.5	79/21	NR	NR	≤T2: 53 ≥T3: 47	≤T2: 52.5 ≥T3: 47.5	26.7 ± 18.6	14.1 ± 11.2
Deimling, 2024^27^	Austria	Retrospective	255^a^	255^a^	66 [13]	71[14]	NR	NR	O: 31.4 ± 28.2		≤T2: 58 ≥T3: 42	≤T2: 63.1 ≥T3: 36.9	24 [17]	15.5 [14]
Dhar, 2008^12^	USA and Switzerland	Prospective	322	336	66.6 ± 9.3	61.4 ± 9	78/22	79/21	NR	NR	NR	NR	22.2 ± 5.7	12.2 ± 5
Gschwend, 2019^28^	Germany	RCT	198	203	66.6 ± 11.2	67.3 ± 8.9	76/24	80/20	O: 40.3^b^		≤T2: 60.1 ≥T3: 39.9	≤T2: 51.7 ≥T3: 48.3	33.5 ± 18.7	19 ± 10.4
Heck, 2025^14^	NR	RCT	NR	NR	NR	NR	NR	NR	NR	NR	NR	NR	NR	NR
Holmer, 2009^29^	Sweden	Retrospective	101	69	65.8 ± 7.4	66.7 ± 8.4	82.2/17.8	81.2/18.8	38.4 ± 11.4	93.6 ± 12.9	≤T2: 52.5 ≥T3: 47.5	≤T2: 66.7 ≥T3: 33.3	37.3 ± 12.6	9.8 ± 7.7
Hugen, 2010^30^	USA	Retrospective	54	206	NR	NR	NR	NR	NR	NR	≤T2: 65 ≥T4: 35		NR	NR
Jensen, 2011^31^	Denmark	Retrospective	265	204	63.7 ± 9	62.7 ± 8.2	78.5/21.3	76.5/23.5	45.5 ± 10.6	113.1 ± 10.4	≤T2: 60.4 ≥T3: 39.6	≤T2: 54.9 ≥T3: 45.1	23.6 ± 9.9	6.2 ± 2.7
Lerner, 2024^6^	USA and Canada	RCT	292	300	69 [37.9]	68 [38.9]	81/19	78/22	O: 73.2^b^		≤T2: 74.7 ≥T3: 25.3	≤T2: 79.2 ≥T3: 28.0	39 (15.9)^c^	24 (6.6)^c^
Mata, 2015^32^	USA and Canada	Retrospective	205	224	O: 62 ± 11.9		O: 81/19		NR	NR	O: ≤T2: 100 ≥T3: 0		37.7 ± 26.1	14.3 ± 6.7
Poulsen, 1998^33^	Denmark	Retrospective	126	68	61 ± 10.3	62.1 ± 7.7	81/19	81/19	24 ± 10.8	58.8 ± 19.2	≤T2: 44.4 ≥T3: 55.6	≤T2: 50.7 ≥T3: 49.3	26.2 ± 11.2	14.5 ± 5.3
Simone, 2016^34^	Italy	Retrospective	349	584	65.4 ± 8.7	66.9 ± 9.2	88.5/11.5	86/14	NR	NR	≤T2: 55 ≥T3: 45	≤T2: 46.5 ≥T3: 53.5	30.4 ± 13.4	19.7 ± 11.1
Wei, 2024^35^	China	Retrospective	80^a^	80^a^	66.8 ± 9.6	66.6 ± 10.6	81.2/18.7	90/10	O: 38.7^b^		≤T2: 71.3 ≥T3: 28.8	≤T2: 71.3 ≥T3: 28.8	16.2 ± 6.4	13.6 ± 6.6

*Note*: Continuous data are presented as mean ± standard deviation, unless otherwise stated as median [IQR]. Binary data are presented as percentages or numbers, as identified.

Abbreviations: CG = control group (standard/limited pelvic lymph node dissection); IG = intervention group (extended pelvic lymph node dissection); IQR = interquartile range; NR = not reported; O = overall (value reported for the entire cohort, not stratified by group); RCT = randomized controlled trial; SD = standard deviation.

^a^ PS‐matched = propensity score–matched cohort.

^b^ Follow‐up reported as median.

^c^ Number of lymph nodes reported as median (range).

### 3.2 Quality assessment and risk of bias

All three RCTs were rated as low risk of bias using RoB 2 (Figure S1). Among observational studies, three were low risk, nine moderate (mainly due to confounding [D1] and selection bias [D3]) and one serious risk (D1, with additional concerns in D3 and D5) (Figure S2).

Across nine outcomes assessed with GRADE, indirectness was not serious. Risk of bias ranged from not serious to serious, and inconsistency and imprecision from not serious to very serious, resulting in overall certainty ranging from very low to moderate.

### 3.3 Pooled analysis

Results are summarized in Table 2.

**TABLE 2:** Summary of analysed outcomes.

Outcomes	Subgroup	Studies (*n*)	Effect (95% CI)	*I* ^2^ (%)	*p*‐value	Favours
Overall survival	RCT	2	HR = 0.98 (0.73 to 1.31)	59%	0.90	NS
	Non‐RCT	5	HR = 0.79 (0.65 to 0.96)	28%	0.02	ePLND
	Global	7	HR = 0.86 (0.72 to 1.03)	50%	0.09	NS
Recurrence‐free survival	RCT	2	HR = 0.96 (0.71 to 1.31)	52%	0.81	NS
	Non‐RCT	10	HR = 0.70 (0.60 to 0.81)	53%	<0.01	ePLND
	Global	12	HR = 0.74 (0.63 to 0.81)	68%	<0.01	ePLND
Cancer‐specific survival	RCT	2	HR = 0.87 (0.52 to 1.45)	79%	0.59	NS
	Non‐RCT	4	HR = 0.66 (0.50 to 0.88)	47%	<0.01	NS
	Global	6	HR = 0.74 (0.56 to 0.97)	71%	0.03	ePLND
5‐years overall survival	RCT	2	RR = 0.99 (0.78 to 1.25)	66%	0.77	NS
	Non‐RCT	4	RR = 0.85 (0.69 to 1.04)	73%	0.86	NS
	Global	6	RR = 0.90 (0.78 to 1.05)	58%	0.17	NS
5‐years recurrence‐free survival	RCT	2	RR = 0.97 (0.81 to 1.17)	34%	0.77	NS
	Non‐RCT	9	RR = 0.75 (0.66 to 0.85)	43%	<0.01	ePLND
	Global	11	RR = 0.79 (0.70 to 0.90)	62%	<0.01	ePLND
5‐years cancer‐specific survival	RCT	1	RR = 0.69 (0.51 to 0.94)	—	0.02	ePLND
	Non‐RCT	1	RR = 1.03 (0.75 to 1.42)	—	0.84	NS
	Global	2	RR = 0.84 (0.57 to 1.25)	68%	0.40	NS
Lymphocele	RCT	2	RR = 2.13 (1.08 to 4.20)	0%	0.03	sPLND
	Non‐RCT	1	RR = 1.08 (0.10 to 11.69)	—	0.95	NS
	Global	3	RR = 2.02 (1.05 to 3.89)	0%	0.03	sPLND
Major complications	30 days	3	RR = 0.84 (0.42 to 1.66)	88%	0.61	NS
	90 days	4	RR = 1.17 (0.99 to 1.39)	0%	0.07	NS
	180 days	1	RR = 0.33 (0.07 to 1.60)	—	0.17	NS
	Global	5	RR = 1.02 (0.74 to 1.41)	66%	0.90	NS
Recurrence site	Local recurrence	6	RR = 0.88 (0.78 to 0.99)	37%	0.04	ePLND
	Distance recurrence	6	RR = 0.55 (0.22 to 1.36)	93%	0.19	NS
	Local‐distance recurrence	4	RR = 0.97 (0.82 to 1.14)	0%	0.69	NS
	Global	7	RR = 0.80 (0.65 to 0.98)	82%	0.03	ePLND

Abbreviations: 95% CI = 95% confidence interval; HR = hazard ratio; *I* ^2^ = *I*‐squared (measure of heterogeneity); NS = not significant; RCT = randomized controlled trial; RR = risk ratio.

### 3.4 Survival outcomes

**FIGURE 2:**
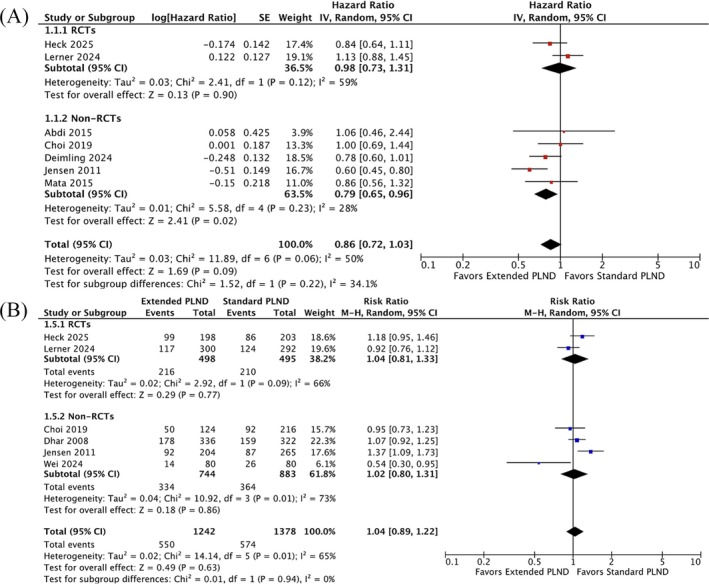
Forest plot evaluating: OS (2A) and 5‐year OS (2B).

OS was evaluated in two RCTs^6^, ^14^ and five non‐RCTs.^23^, ^25^, ^27^, ^31^, ^32^ No difference was observed in RCTs between ePLND and sPLND (HR = 0.98; 95% CI 0.73–1.31; *p* = 0.90; *I* ^2^ = 59%), whereas non‐RCTs favoured ePLND (HR = 0.79; 95% CI 0.65–0.96; *p* = 0.02; *I* ^2^ = 28%). Overall, no significant difference was observed (HR = 0.86; 95% CI 0.72–1.03; *p* = 0.09; *I* ^2^ = 50%), with no subgroup difference (*p* = 0.22) (Figure 2A).

**FIGURE 3:**
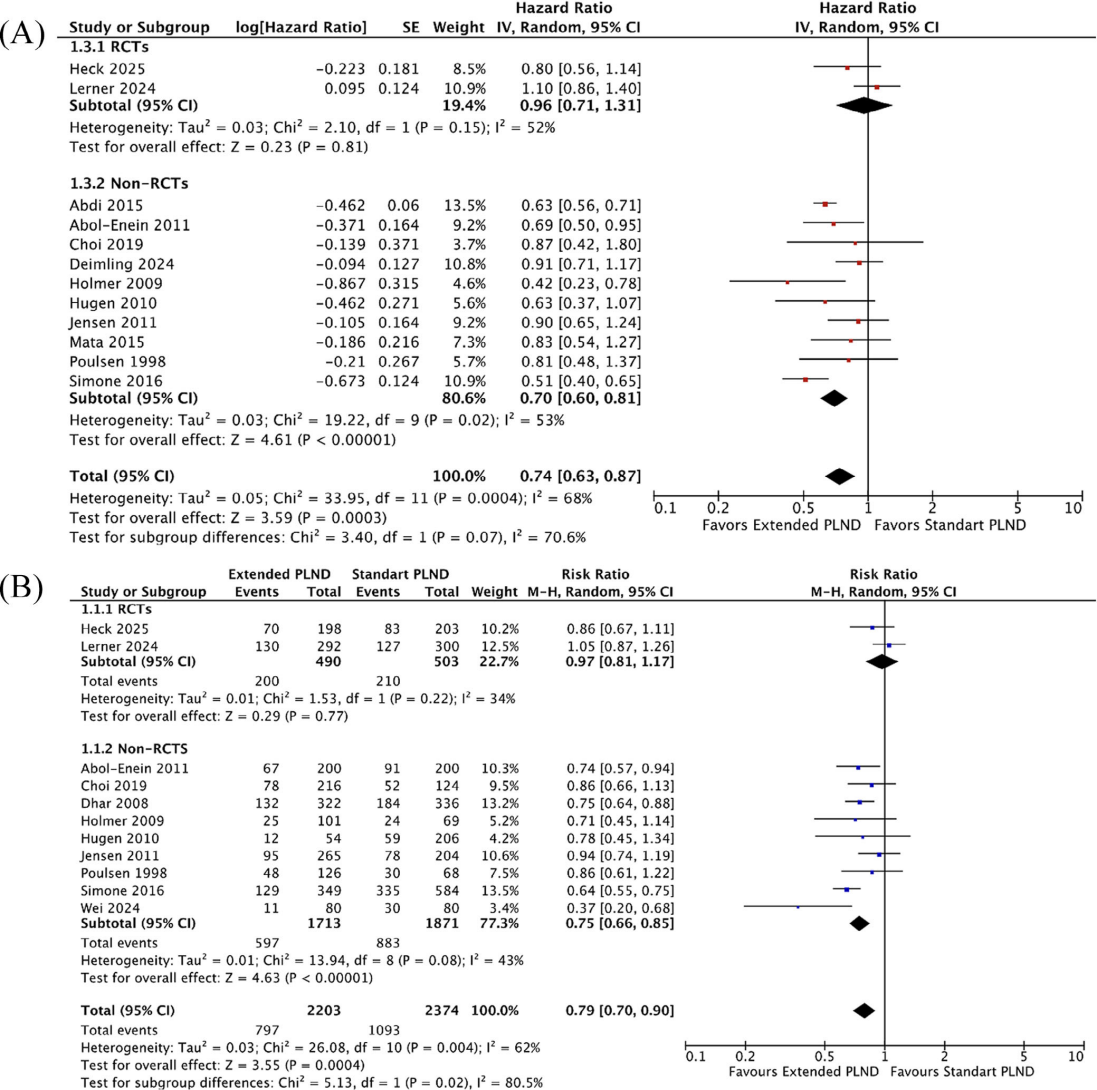
Forest plot evaluating: RFS (3A) and 5‐year RFS (3B).

Twelve studies reported RFS, including two RCTs^6^, ^14^ and 10 non‐RCTs.^23^, ^24^, ^25^, ^27^, ^29^, ^30^, ^31^, ^32^, ^33^, ^34^ RCTs showed no difference (HR = 0.96; 95% CI 0.71–1.31; *p* = 0.81; *I* ^2^ = 52%), whereas non‐RCTs favoured ePLND (HR = 0.70; 95% CI 0.60–0.81; *p* < 0.001; *I* ^2^ = 53%). Overall, ePLND improved RFS (HR = 0.74; 95% CI 0.63–0.87; *p* = 0.0003; *I* ^2^ = 68%), with no subgroup difference (*p* = 0.07) (Figure 3A).

**FIGURE 4:**
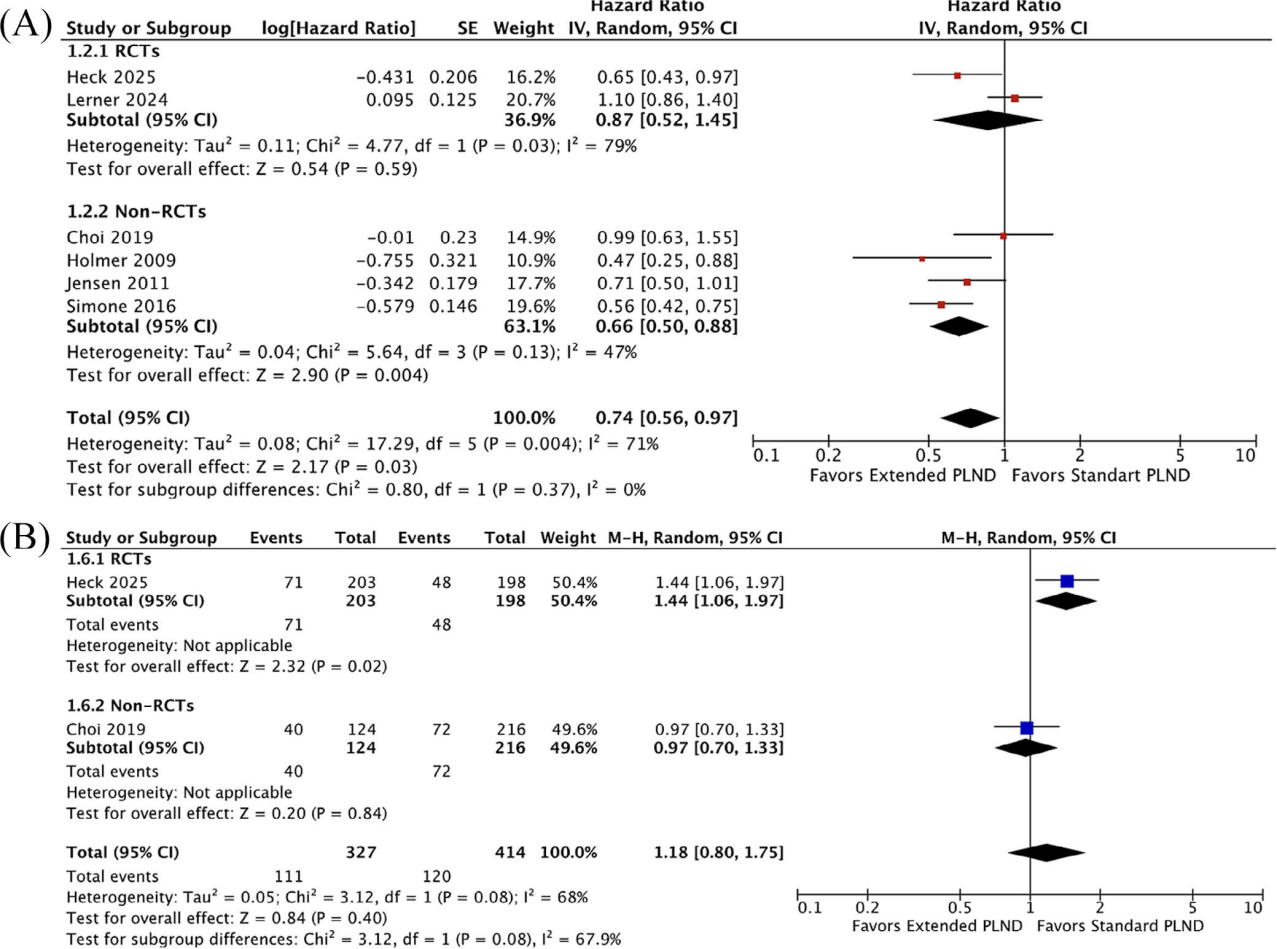
Forest plot evaluating: CSS (4A) and 5‐year CCS (4B).

CSS was analysed in two RCTs,^6^, ^14^ and four non‐RCTs^29^, ^31^, ^34^, ^35^ were included. RCTs showed no difference (HR = 0.87; 95% CI 0.52–1.45; *p* = 0.59; *I* ^2^ = 79%), while non‐RCTs favoured ePLND (HR = 0.66; 95% CI 0.50–0.88; *p* = 0.004; *I* ^2^ = 47%). Overall, ePLND improved CSS (HR = 0.74; 95% CI 0.56–0.97; *p* = 0.03; *I* ^2^ = 71%), with no subgroup difference (*p* = 0.37) (Figure 4A).

### 3.5 Five‐year survival outcomes

For 5‐year OS analysis, two RCTs^6^, ^14^ and four non‐RCTs^12^, ^25^, ^31^, ^35^ were included. No differences were observed in RCTs (RR = 0.99; 95% CI 0.78–1.25; *p* = 0.77; *I* ^2^ = 61%) or non‐RCTs (RR = 0.85; 95% CI 0.69–1.04; *p* = 0.12; *I* ^2^ = 62%). Overall, no difference was found (RR = 0.90; 95% CI 0.78–1.05; *p* = 0.17; *I* ^2^ = 58%), with no subgroup difference (*p* = 0.34) (Figure 2B).

Five‐year RFS was evaluated in 11 studies (*n* = 4577), including two RCTs^6^, ^14^ and nine non‐RCTs.^12^, ^24^, ^25^, ^29^, ^30^, ^31^, ^33^, ^34^, ^35^ RCTs showed no difference between ePLND and sPLND (RR = 0.97; 95% CI 0.81–1.17; *p* = 0.77; *I* ^2^ = 34%), whereas non‐RCTs favoured ePLND (RR = 0.75; 95% CI 0.66–0.85; *p* < 0.001; *I* ^2^ = 43%). Overall, ePLND improved 5‐year RFS (RR = 0.79; 95% CI 0.70–0.90; *p* < 0.001; *I* ^2^ = 62%), with a significant subgroup difference (*p* = 0.02) (Figure 3B).

Five‐year CSS was evaluated in one RCT^14^ and one non‐RCT.^25^ No difference was observed between ePLND and sPLND (RR = 0.84; 95% CI 0.57–1.25; *p* = 0.40; *I* ^2^ = 68%), with no subgroup difference (*p* = 0.08) (Figure 4B).

### 3.6 Perioperative and oncologic outcomes

**FIGURE 5:**
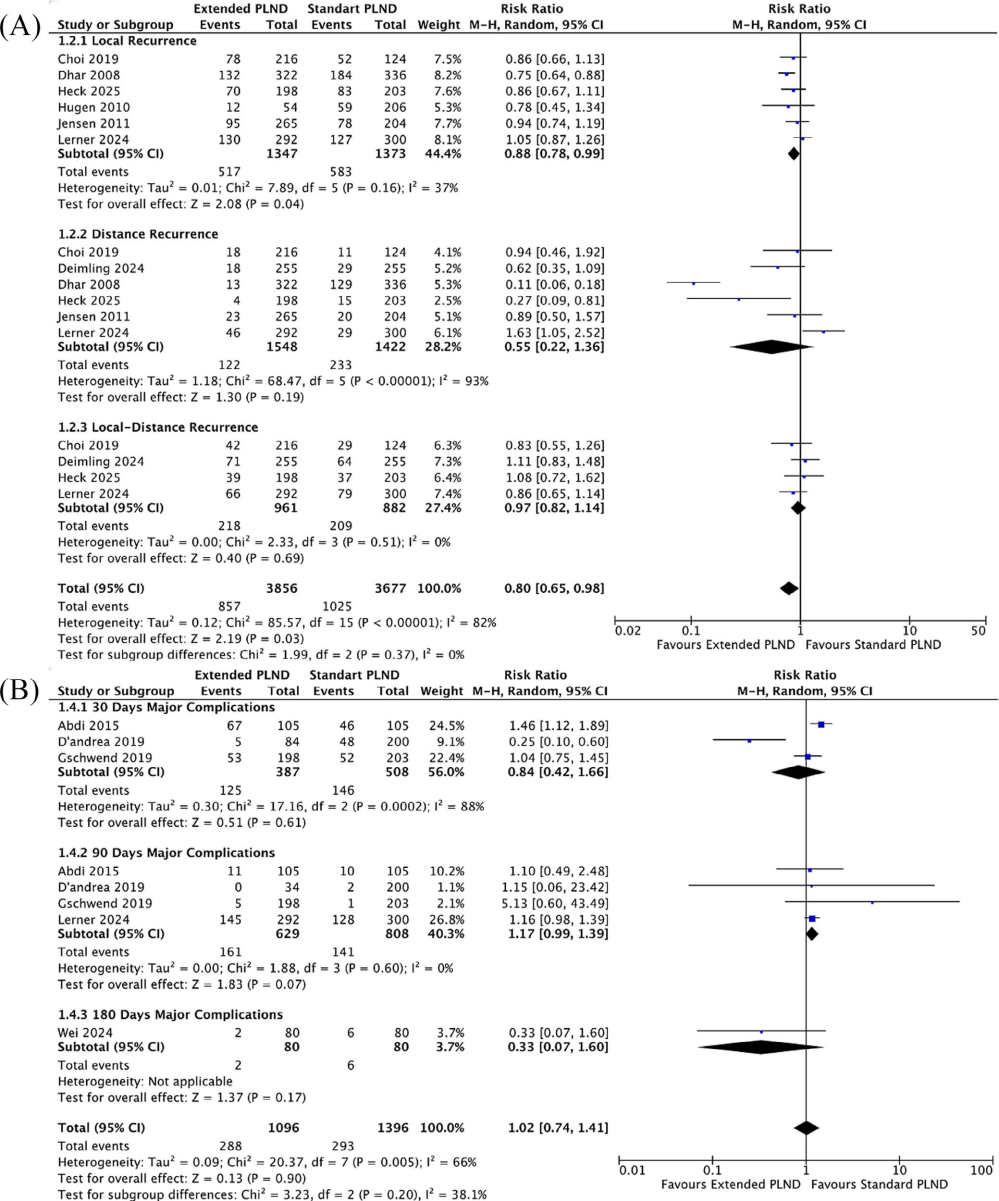
Forest plot evaluating: recurrence site (5A) and major complications (5B).

Seven studies^6^, ^12^, ^14^, ^25^, ^27^, ^30^, ^31^ evaluated recurrence patterns. ePLND was associated with lower local recurrence (RR = 0.88; 95% CI 0.78–0.99; *p* = 0.04; *I* ^2^ = 37%), with no difference in distant (RR = 0.55; 95% CI 0.22–1.36; *p* = 0.19; *I* ^2^ = 93%) or combined recurrence (RR = 0.97; 95% CI 0.82–1.14; *p* = 0.69; *I* ^2^ = 0%). Overall, recurrence was higher in the sPLND group (RR = 0.80; 95% CI 0.65–0.98; *p* = 0.03; *I* ^2^ = 82%), with no subgroup difference (*p* = 0.37) (Figure 5A).

At 30 days, major complications assessed in three studies^23^, ^26^, ^28^ showed no difference (RR = 0.84; 95% CI 0.42–1.66; *p* = 0.61; *I* ^2^ = 88%). At 90 days, four studies^6^, ^23^, ^26^, ^28^ showed a non‐significant trend favouring ePLND (RR = 1.17; 95% CI 0.99–1.39; *p* = 0.07; *I* ^2^ = 0%). At 180 days, one study^32^ showed no difference (RR = 0.33; 95% CI 0.07–1.60; *p* = 0.17). Overall, no difference was observed (RR = 1.02; 95% CI 0.74–1.41; *p* = 0.90; *I* ^2^ = 66%), with no subgroup difference (*p* = 0.20) (Figure 5B).

Two RCTs^6^, ^28^ and one non‐RCT^33^ were included in the lymphocele analysis. RCTs showed higher lymphocele incidence with ePLND (RR = 2.13; 95% CI 1.08–4.20; *p* = 0.03; *I* ^2^ = 0%), while the non‐RCT showed no difference. Overall, lymphocele was less frequent with sPLND (RR = 2.02; 95% CI 1.05–3.89; *p* = 0.03; *I* ^2^ = 0%), with no subgroup difference (*p* = 0.59) (Figure S3).

### 3.7 Sensitivity and additional analyses

Sensitivity analyses showed stable results across outcomes, with no meaningful changes in effect estimates after sequential exclusion of individual studies. For OS, key studies^6^, ^31^ contributed most to heterogeneity, but their exclusion did not alter the overall interpretation. No relevant publication bias was identified.

Meta‐regression did not demonstrate significant effect modification for any evaluated covariate, including study design, neoadjuvant therapy, tumour stage, follow‐up or lymph node yield. Detailed sensitivity and meta‐regression results are provided in the Supporting Information (Figures S4–S7).

## 4 DISCUSSION

This systematic review and meta‐analyses including 5960 participants showed that ePLND did not provide a statistically significant OS benefit compared with sPLND in patients undergoing radical cystectomy. Although observational studies suggested an advantage, this was not reproduced in RCTs, indicating that the apparent benefit may reflect biases inherent to non‐randomized designs.

This divergence is well described in surgical oncology.^36^, ^37^ Patients undergoing more extensive dissections are often treated at high‐volume centres or by experienced surgeons, factors independently associated with improved outcomes.^38^, ^39^, ^40^ In addition, removal of more lymph nodes increases detection of micrometastases (stage migration), potentially improving survival estimates without altering disease course.^23^, ^25^, ^27^, ^31^, ^32^

Consistently, 5‐year OS showed no advantage with ePLND. Although some observational studies suggested benefit, this disappeared in RCTs, reinforcing the role of residual confounding. Similarly, the apparent CSS benefit did not persist in randomized data, with the positive finding from Heck et al.^14^ not replicated by Lerner et al.^6^ Increased nodal detection may instead improve patient selection for systemic therapies rather than confer direct survival benefit.^41^, ^42^, ^43^

RFS showed a significant overall effect favouring ePLND; however, this was driven by observational studies, as RCTs showed no difference. This pattern further supports the influence of non‐randomized biases rather than a true therapeutic effect.

Recurrence‐site analysis yielded inconsistent findings across RCTs. While Lerner et al.^6^ suggested reduced distant recurrence with ePLND, Heck et al.^14^ reported the opposite, contributing to heterogeneity. For local recurrence, the observed difference was small and not significant in RCT‐only analyses. Overall, these findings suggest that the extent of lymph node dissection is unlikely to be a primary determinant of oncological control.

From a perioperative perspective, ePLND was associated with higher lymphocele rates, likely due to more extensive lymphatic disruption.^44^ However, major complications was similar between techniques, being more strongly influenced by patient‐related factors such as age, BMI, and comorbidities.^45^, ^46^

This study has limitations that should be considered when interpreting the findings. First, the analysis included a combination of RCTs and observational studies, which may introduce methodological heterogeneity. Second, some variability existed in the surgical templates used to define standard and extended lymph node dissection across studies, which may limit direct comparability between interventions. Finally, the heterogeneity observed in some outcomes suggests that additional factors—such as variability in surgical experience, the use of perioperative systemic therapies, and tumour biology—may influence oncological outcomes after radical cystectomy.

Future studies should identify subgroups that may benefit from extended dissection and clarify its role in the context of modern systemic therapies, including neoadjuvant chemotherapy and immunotherapy. Additional large RCTs with standardized templates are needed to better define the therapeutic impact of lymph node dissection in bladder cancer.

## 5 CONCLUSION

In this updated systematic review and meta‐analysis of 5960 patients from 16 studies, including contemporary RCTs, ePLND did not improve OS compared with sPLND in patients undergoing radical cystectomy. The apparent benefit observed in observational studies was not confirmed in randomized analyses. Although pooled data suggested improved recurrence‐related outcomes, including overall and 5‐year recurrence, these effects were largely driven by non‐randomized evidence. Overall, current evidence does not support the routine use of ePLND solely to improve survival. Further high‐quality RCTs are needed to clarify its role.

## AUTHOR CONTRIBUTIONS

Rafael Matos Vieira Gordilho, Nilo Jorge Carvalho Leão Barreto, Rodrigo Barbosa Freire Silvão, Felipe Pereira Garrido Pazos, Pedro Rodrigues Queiroz, Matheus Oliveira Figueiredo, Ricardo Andrade Freitas Souza, João Pedro Gomes da Conceição Oliva, Luma Gabriella Firme Sampaio Góes, Rafael Cintra Rosa Corrêa Oliveira, Lorena Barboza de Sousa, Felipe Pinho Albuquerque Silva, Leonardo Marques Calazans, Wendel Souza Kruschewsky, Alexandre Azevedo Ziomkowski, Eduardo Deda Mendonça Filho, Gabriel Silva Rocha, Marcos Tobias‐Machado, and Raphael Brandão Moreira contributed to the conception and design of the study, data acquisition, analysis and interpretation of data, drafting and critical revision of the manuscript, approved the final version for publication, and agree to be accountable for all aspects of the work.

## CONFLICT OF INTEREST STATEMENT

The authors declare that they have no competing interests.

## Supporting information


**Table S1.** Clinicopathological characteristics of the included participants.

**Figure S1.** Risk of bias (RoB 2) of randomized studies.

**Figure S2.** Risk of bias (ROBINS‐I) of non‐randomized studies.

**Figure S3.** Forest plot evaluating: lymphocele.

**Figure S4.** Baujat plot of the OS outcome.

**Figure S5.** LOO analysis of OS (S5A) and RFS (S5B).

**Figure S6.** LOO analysis of 5‐year OS (S6A) and 5‐year RFS (S6B).

**Figure S7.** LOO analysis of major complications (S7A) and publication bias (S7B).

## Data Availability

All data analysed in this study are included in the published articles and Supporting Information.
