# Supplementary material for: Oncological outcomes of extended versus standard pelvic lymph node dissection in radical cystectomy: An updated systematic review and meta‐analysis
Source: BJUI Compass. 2026 Aug 2;7(8):e70257. doi: 10.1002/bco2.70257 (PMC13430057; doi:10.1002/bco2.70257)
Supplement: Supplementary file 1 — Table S1. Clinicopathological characteristics of the included participants. [file BCO2-7-e70257-s005.docx]

**Supplementary material**

**Search strategy**

Pubmed: ("Urinary Bladder Neoplasms" OR "bladder cancer" OR "bladder carcinoma" OR "urothelial carcinoma" OR "transitional cell carcinoma") AND ("Cystectomy" OR "radical cystectomy") AND ("Lymph Node Excision" OR "lymph node dissection" OR "pelvic lymph node dissection" OR PLND OR ePLND OR sPLND OR "extended lymph node dissection" OR "standard lymph node dissection")

Embase: ('bladder cancer'/exp OR 'bladder tumor' OR 'urothelial carcinoma' OR 'transitional cell carcinoma') AND ('cystectomy'/exp OR 'radical cystectomy') AND ('lymph node dissection'/exp OR 'pelvic lymph node dissection' OR PLND OR ePLND OR sPLND OR 'extended lymph node dissection' OR 'standard lymph node dissection')

Cochrane Library: ("bladder cancer" OR "bladder carcinoma" OR "urothelial carcinoma" OR "transitional cell carcinoma") AND ("radical cystectomy") AND ("lymph node dissection" OR "pelvic lymph node dissection" OR PLND OR ePLND OR sPLND OR "extended lymph node dissection" OR "standard lymph node dissection")

Web of Science: ("bladder cancer" OR "bladder carcinoma" OR "urothelial carcinoma" OR "transitional cell carcinoma") AND ("radical cystectomy") AND ("lymph node dissection" OR "pelvic lymph node dissection" OR PLND OR ePLND OR sPLND OR "extended lymph node dissection" OR "standard lymph node dissection")

**Sensitivity and additional analyses**

The Baujat plot for OS (Supplementary Figure 4) showed that Jensen 2011 and Lerner 2024 contributed most to the overall heterogeneity, appearing on the right side of the plot. Both had a notable influence on the pooled estimate, particularly Lerner 2024, which exerted a larger effect on the magnitude of the estimate. The remaining studies showed limited contributions to both heterogeneity and the summary effect.

Visual inspection of the funnel plot for OS (Supplementary Figure 7B) showed a relatively symmetrical distribution of studies around the central estimate (log hazard ratio). The RCTs clustered toward the upper portion of the plot, reflecting lower standard error and greater precision. The non-RCTs were more dispersed at the base of the funnel, consistent with smaller sample sizes and greater methodological variability. Despite differences in precision, we did not identify a directional asymmetry pattern suggesting substantial publication bias.

In the LOO sensitivity analysis, sequential exclusion of individual studies did not substantially change the pooled estimate. The HR ranged from 0.79 to 0.92, without meaningful impact on the overall statistical significance. The exclusion of Lerner 2021 provided a significant effect favorable to ePLND (HR = 0.79; 95% CI 0.68–0.92; I² = 14,1%). Furthermore, the exclusion of Jensen 2011 increased the HR to 0.92 (95% CI 0.81–1.05) and reduced heterogeneity to 0%, withhese findings indicate stability of the pooled estimate. (Supplementary Figure 5A).

For RFS, sequential exclusion of individual studies did not significantly alter the pooled effect estimate or heterogeneity. The HR ranged from 0.71 to 0.78, consistently favoring ePLND without changes in the direction of the effect. These findings suggest robustness of the analysis and absence of excessive influence from individual studies (Supplementary Figure 5B).

In the sensitivity analysis for 5-year OS, sequential exclusion of studies did not change the direction of the pooled effect. The RR ranged from 0.87 to 0.96, and the confidence intervals consistently crossed the null value. Also, the exclusion of Lerner 2021 provided a significant effect favorable to ePLND (RR = 0.87; 95% CI 0.75–0.99; I² = 49,2%).Heterogeneity remained high across scenarios, suggesting that variability was not attributable to a single study. (Supplementary Figure 6A).

For 5-year RFS, sequential exclusion of studies did not meaningfully change the pooled estimate. The RR ranged from 0.76 to 0.83, maintaining statistical significance in all scenarios and consistently favoring ePLND. Heterogeneity ranged from moderate after exclusion of Simone 2016 (I² = 47.7%) to 65.5% after omission of Abol-Enein 2011, without major changes in interpretation (Supplementary Figure 6B).

In the sensitivity analysis for major complications, sequential exclusion of studies resulted in RR values ranging from 0.83 to 1.13. Exclusion of D’Andrea 2019 (30-day subgroup) substantially reduced heterogeneity (I² from 65.6% to 18.3%) and yielded a statistically significant estimate favoring sPLND (RR = 0.83; 95% CI 0.70–0.99) (Supplementary Figure 7A).

**Meta-regression**

Meta-regression analyses were performed to explore potential sources of heterogeneity. Regarding study design (RCT vs. non-RCT; k=7), RCTs showed no benefit of ePLND (HR=0.98; 95% CI 0.75–1.29; p=0.898), whereas non-RCTs suggested a benefit (HR=0.79; 95% CI 0.64–0.98); however, no significant effect modification was observed (QM(1)=1.50; p=0.22). Residual heterogeneity was moderate (I²=41.2%; τ²=0.0206), with R²=27.6%.

Regarding year of publication (k=7), a significant average effect was observed (HR=0.84; 95% CI 0.71–0.99; p=0.0358), although the moderator was not significant (p=0.16), indicating no temporal effect modification. Residual heterogeneity remained moderate (I²=39.2%; τ²=0.0184), with R²=35.2%, suggesting a non-significant trend toward reduced benefit in more recent studies.

Regarding neoadjuvant therapy (k=6), no association with treatment effect was observed (β=0.0143; p=0.79), with no heterogeneity explained (R²=0%) and persistent residual heterogeneity (I²=65.1%). Similarly, regarding advanced pT stage (≥T3; k=7), no association with outcomes was observed (β=0.0005; p=0.93; R²=0%; I²=58.4%).

Regarding follow-up time (k=7), no significant effect modification was observed (β=−0.0020; p=0.71; R²=0%; I²=56.8%). Likewise, regarding the number of lymph nodes removed (k=7), no association with outcomes was observed (β=−0.0063; p=0.80; R²=0%; I²=57.4%).

**Tables and their legends**

| **Author, Year** | **pN stage, n (%): ePLND** | **pN stage, n (%): sPLND** | **Histology (UC/NUH), n (%): ePLND** | **Histology (UC/NUH), n (%): sPLND** | **Neoadjuvant therapy, n (%):**  **ePLND / sPLND** | **Adjuvant chemotherapy, n (%): ePLND / sPLND** |
| --- | --- | --- | --- | --- | --- | --- |
| Abdi, 2015 | ≤ N0: 87 (82.9) ≥ N1: 18 (17.2) | ≤ N0: 91 (86.7)  ≥ N1: 14 (13.3) | 100 (100) /  0 (0) | 100 (100) /  0 (0) | 30 (28.6) /  20 (19.1) | NR |
| Abol-Enein, 2011 | ≤ N0: 152 (76)  ≥ N1: 48 (24) | ≤ N0: 152 (76)  ≥ N1: 48 (24) | 115 (57.5) /  85 (42.5) | 89 (44.5) /  111 (55.5) | 0 / 0 | 0 / 0 |
| Choi, 2019 | ≤ N0: 0 (0.0)  ≥ N1: 89 (41.2) | ≤ N0: 23 (18.5)  ≥ N1: 0 (0.0) | 216 (100) /  0 (0) | 119 (96) /  5 (4) | 0 / 0 | 65 (30.4) /  23 (18.9) |
| D`Andrea, 2019 | ≤ N0: 25 (73.5)  ≥ N1: 9 (26.5) | ≤ N0: 143 (71.5)  ≥ N1: 57 (28.5) | 22 /  1  VH: 11 | 175 /  6  VH: 15 | 2 (1) /  2 (5.9) | NR |
| Deimling, 2024 | ≤ N0: 124 (49)  ≥ N1: 131 (51) | ≤ N0: 94 (37)  ≥ N1: 161 (63.1) | 466 (86) /  0 (0)  VH: 77 (14) | 336 (86) /  0 (0)  VH: 54 (14) | 106 (42) /  102 (40) | 35 (14) /  36 (14) |
| Dhar, 2008 | ≤ N0: 239 (74)  ≥ N1: 83 (26)) | ≤ N0: 292 (87)  ≥ N1: 44 (13) | 100 (100) /  0 (0) | 100 (100) /  0 (0) | 0 / 0 | NR |
| Gschwend, 2019 | ≤ N0: 152 (77)  ≥ N1: 44 (22) | ≤ N0: 147 (72)  ≥ N1: 56 (28) | NR | NR | 0 / 0 | 28 (14) /  30 (15) |
| Heck, 2025 | NR | NR | NR | NR | NR | NR |
| Holmer, 2009 | ≤ N0: 63 (62)  ≥ N1: 38 (38) | ≤ N0: 57 (83)  ≥ N1: 12 (18) | 100 (100) /  0 (0) | 100 (100) /  0 (0) | 0 / 0 | 16 (16) /  9 (13) |
| Hugen, 2010 | NR | NR | NR | NR | NR | NR |
| Jensen, 2011 | ≤ N0: 204 (77)  ≥ N1: 61 (23) | ≤ N0: 161 (79)  ≥ N1: 43 (21) | 256 (97) /  9 (3) | 194 (95) /  10 (5.0) | 0 / 0 | 0 / 0 |
| Lerner, 2019 | ≤ N0: 217 (74)  ≥ N1: 75 (26) | ≤ N0: 229 (76)  ≥ N1: 71 (24) | 252 (86) /  0 (0)  VH: 40 (14) | 264 (88) /  0 (0)  VH: 36 (12) | 166 (57) / 170 (57) | (11) / (11) |
| Mata, 2015 | G: N0: 440 (100)  ≥ N1: 0 (0) | | 100 (100) /  0 (0) | 100 (100) /  0 (0) | NR | NR |
| Poulsen, 1998 | ≤ N0: 90 (71.4)  ≥ N1: 36 (28.6) | ≤ N0: 53 (77.9)  ≥ N1: 15 (22.1) | 124 (98.4) /  2 (1.6) | 67 (98.5) /  1 (1.5) | 0 / 0 | 0 / 0 |
| Simone, 2016 | ≤ N0: 242 (69.3)  ≥ N1: 107 (30.7) | ≤ N0: 397 (68)  ≥ N1: 187 (32.) | 100 (100) /  0 (0) | 100 (100) /  0 (0) | 0 / 0 | 38 (10.9) /  63 (10.8) |
| Wei, 2024 | ≤ N0: 67 (83.7)  ≥ N1: 13 (16.2) | ≤ N0: 64 (80)  ≥ N1: 15 (18.7) | 125 (85.0) /  0 (0)  VH: 22 (15.0) | 100 (77.5) /  0 (0)  VH: 29 (22.5) | 10 (12.5) /  8 (10) | NR |

IG = Intervention group (extended pelvic lymph node dissection); CG = Control group (standard or limited pelvic lymph node dissection); pT = Pathologic tumor stage; pN = Pathologic nodal stage; LN = Lymph node; UC = Urothelial carcinoma; VH = Variant histology (urothelial carcinoma with variant differentiation); NUH = Non-urothelial histology (pure non-urothelial carcinoma subtype); SD = Standard deviation; IQR = Interquartile range; NR = Not reported; ≥N1 = Presence of nodal metastasis; N0 = Absence of nodal metastasis.

Supplementary Table 1. Clinicopathological characteristics of the included participants
