# Supplementary figures and images for: Oncological outcomes of extended versus standard pelvic lymph node dissection in radical cystectomy: An updated systematic review and meta‐analysis
Source: BJUI Compass. 2026 Aug 2;7(8):e70257. doi: 10.1002/bco2.70257 (PMC13430057; doi:10.1002/bco2.70257)

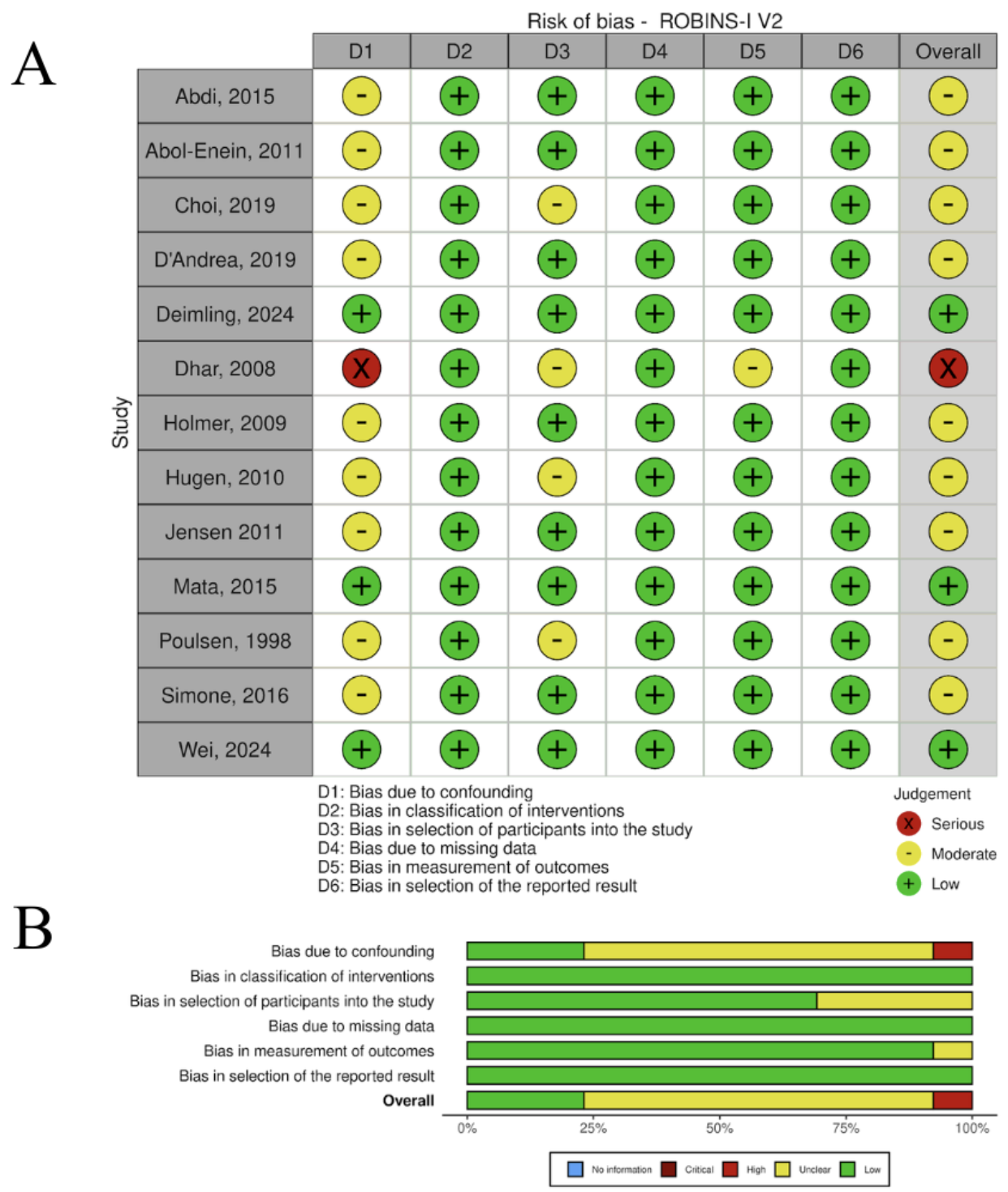

Supplement: Supplementary file 2 — Figure S1. Risk of bias (RoB 2) of randomized studies. [file BCO2-7-e70257-s002.png]

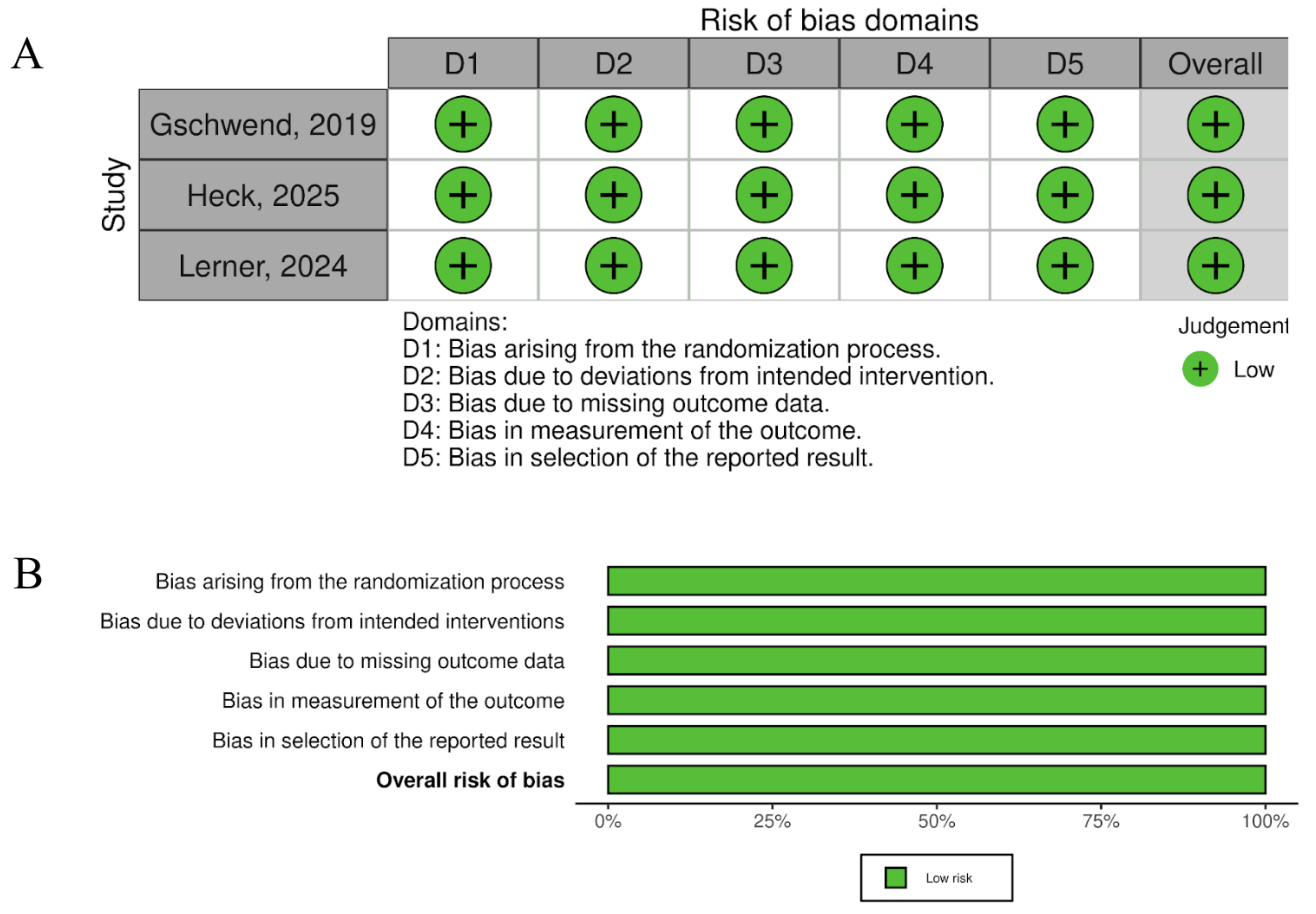

Supplement: Supplementary file 3 — Figure S2. Risk of bias (ROBINS‐I) of non‐randomized studies. [file BCO2-7-e70257-s007.png]

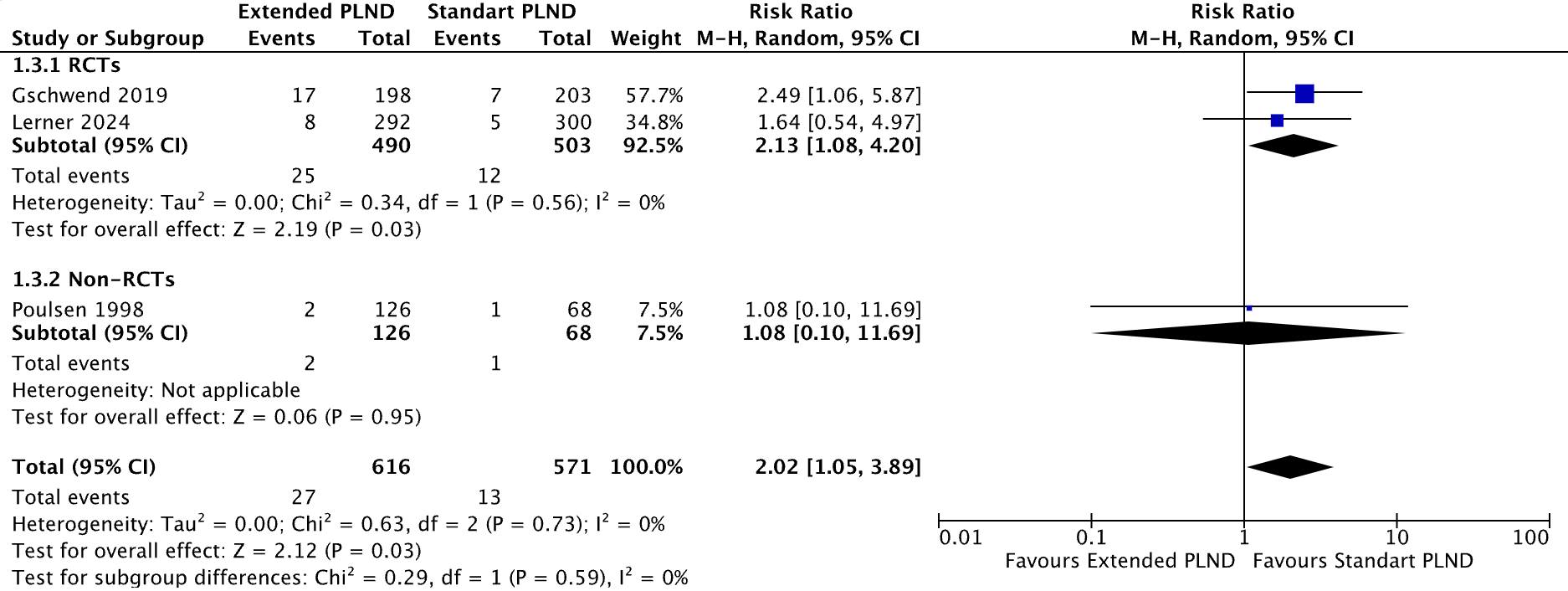

Supplement: Supplementary file 4 — Figure S3. Forest plot evaluating: lymphocele. [file BCO2-7-e70257-s008.png]

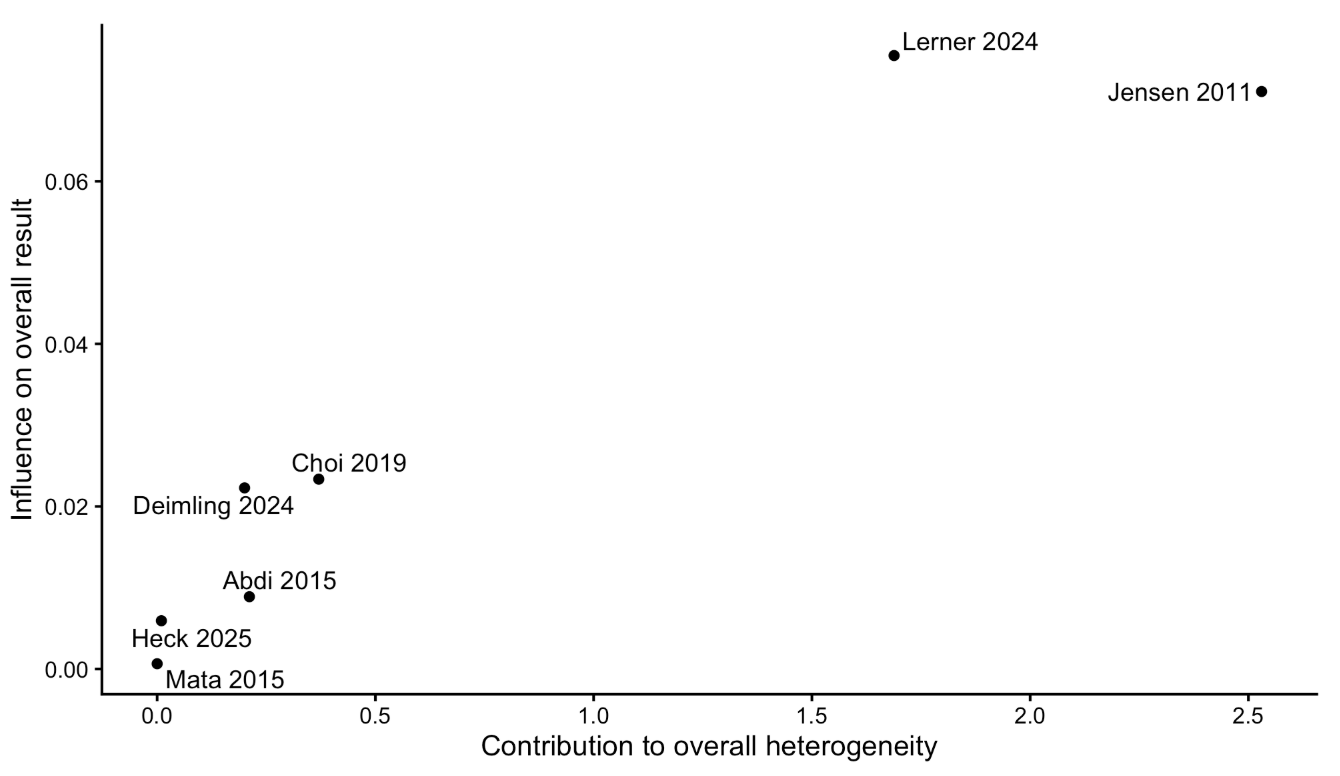

Supplement: Supplementary file 5 — Figure S4. Baujat plot of the OS outcome. [file BCO2-7-e70257-s006.png]

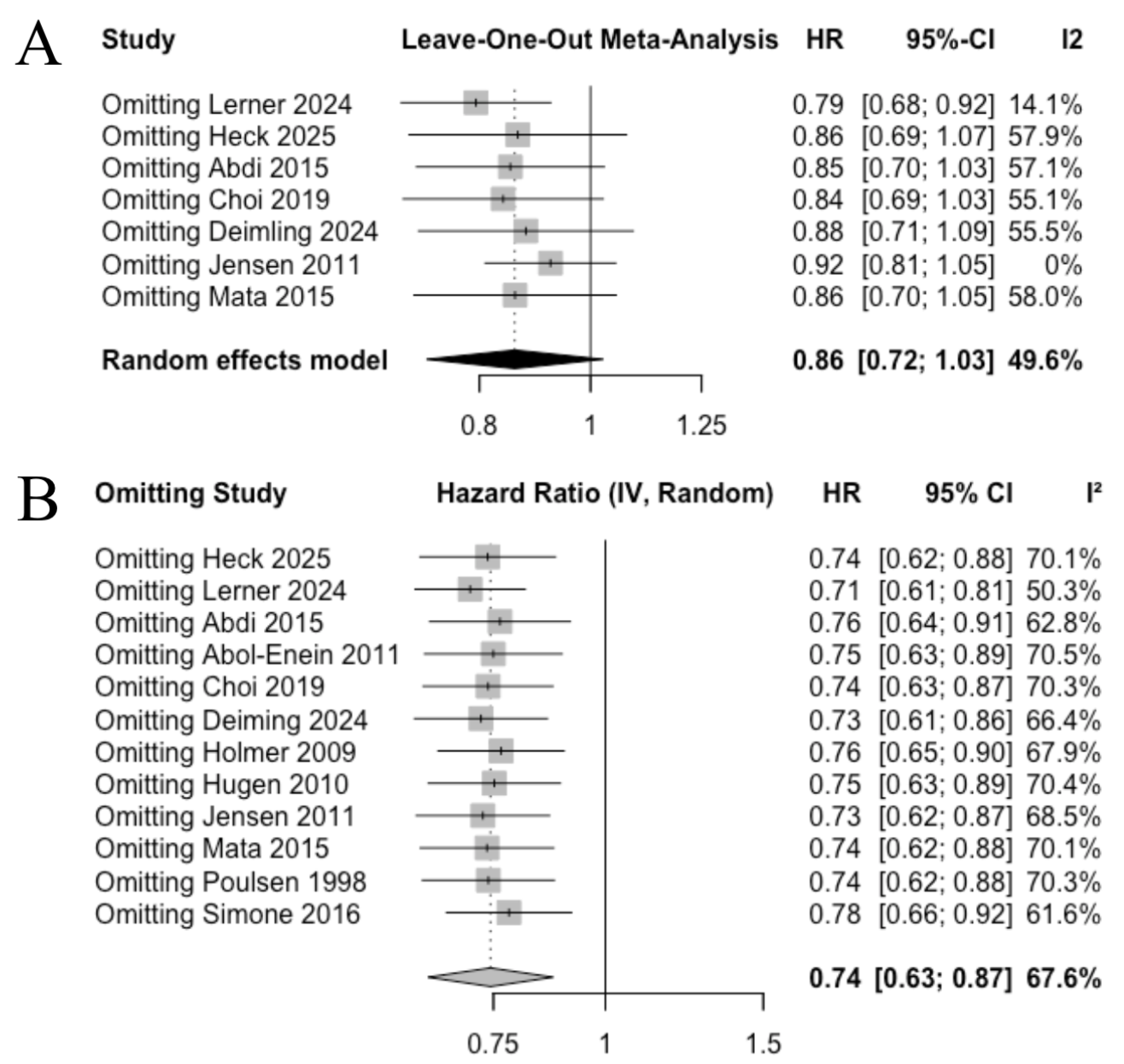

Supplement: Supplementary file 6 — Figure S5. LOO analysis of OS (S5A) and RFS (S5B). [file BCO2-7-e70257-s003.png]

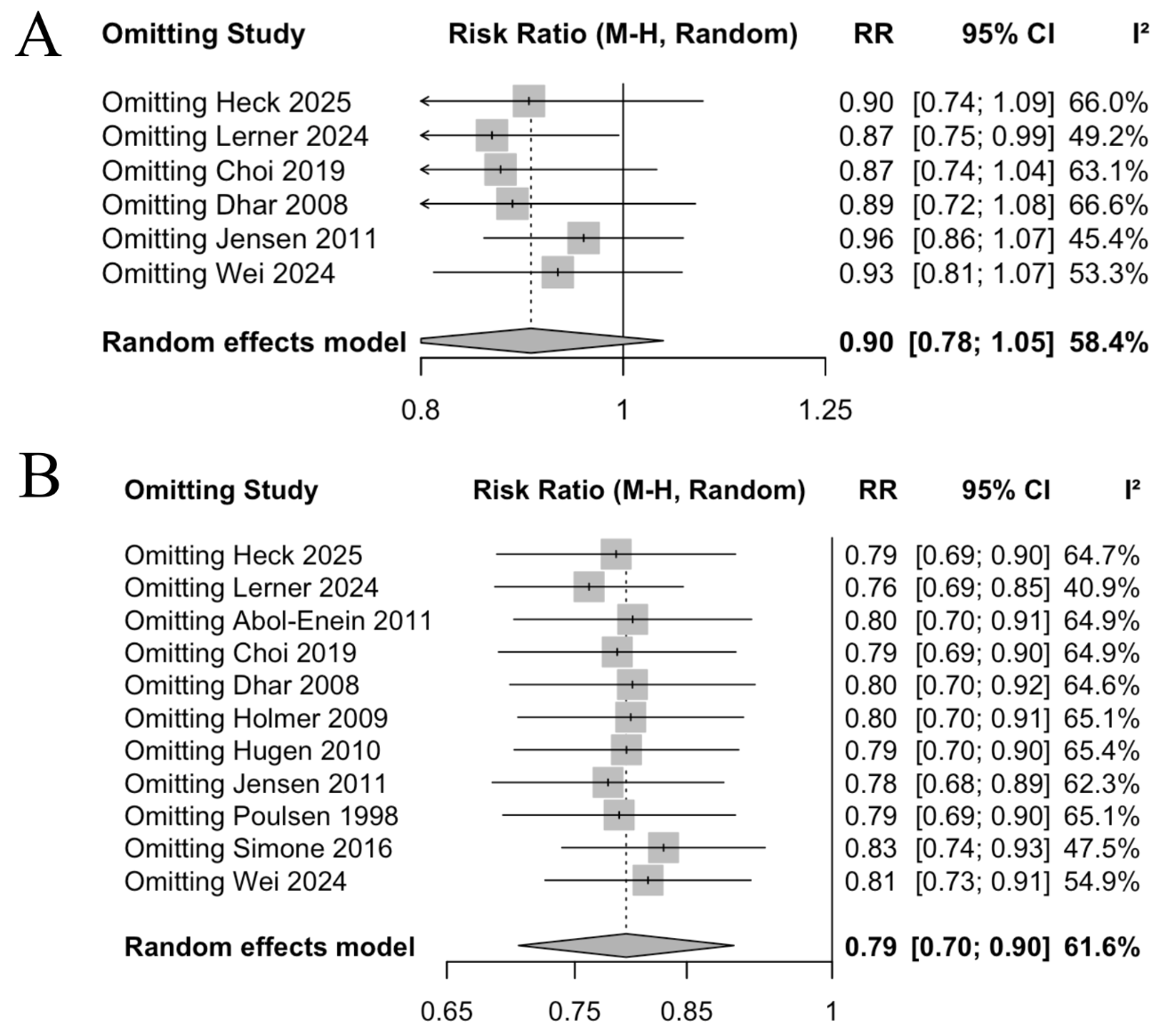

Supplement: Supplementary file 7 — Figure S6. LOO analysis of 5‐year OS (S6A) and 5‐year RFS (S6B). [file BCO2-7-e70257-s001.png]

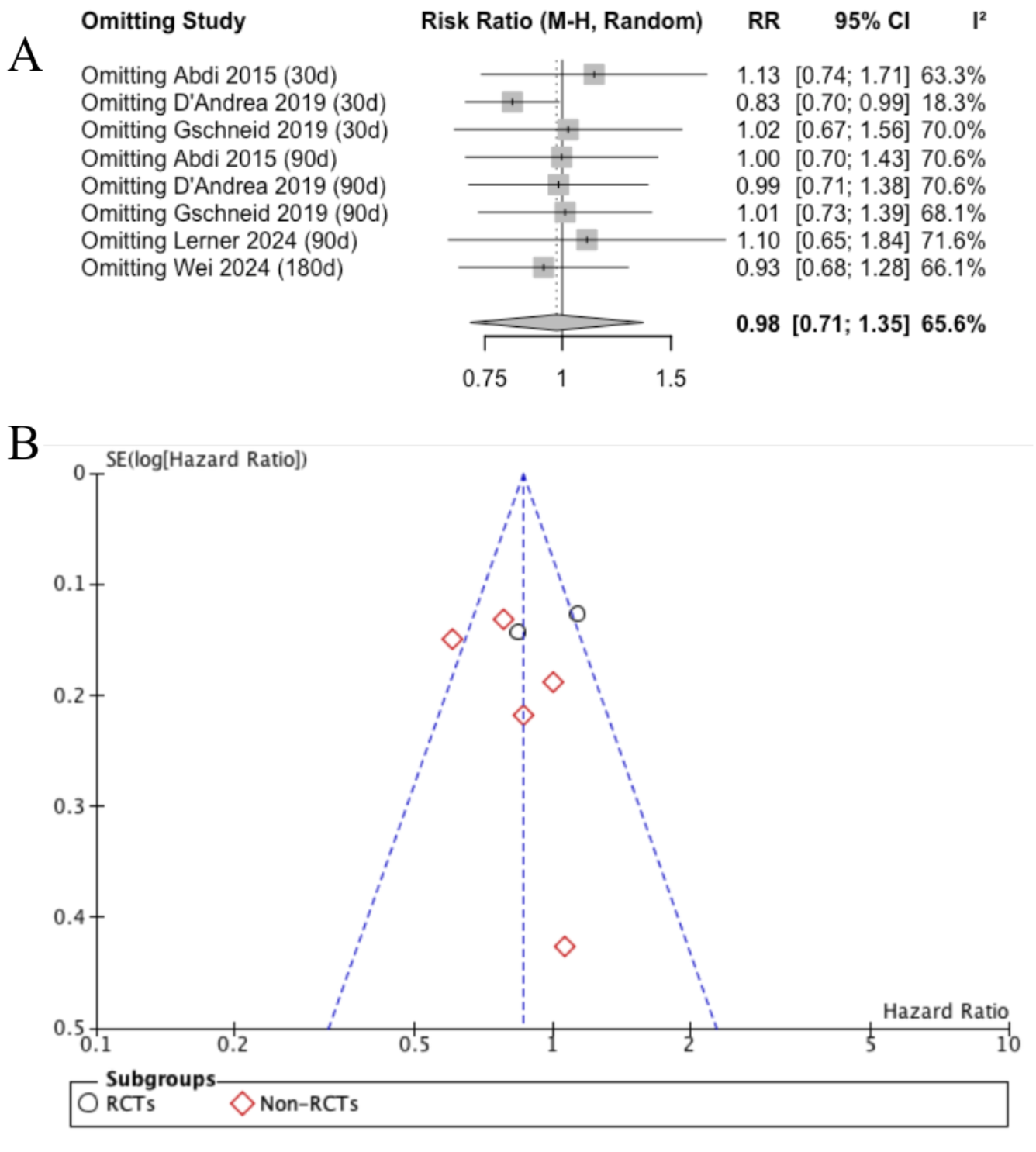

Supplement: Supplementary file 8 — Figure S7. LOO analysis of major complications (S7A) and publication bias (S7B). [file BCO2-7-e70257-s004.png]
